# Feasibility and Acceptability of a Web-Based Caregiver Decision Aid (Safety in Dementia) for Firearm Access: Pilot Randomized Controlled Trial

**DOI:** 10.2196/30990

**Published:** 2021-09-22

**Authors:** Marian E Betz, Evan Polzer, Kathryn Nearing, Christopher E Knoepke, Rachel L Johnson, Lauren Meador, Daniel D Matlock

**Affiliations:** 1 Department of Emergency Medicine University of Colorado Anschutz Medical Campus Aurora, CO United States; 2 Veterans Affairs Eastern Colorado Geriatric Research Education and Clinical Center Rocky Mountain Regional Veterans Affairs Medical Center Aurora, CO United States; 3 Division of Geriatrics and Multidisciplinary Center on Aging University of Colorado Anschutz Medical Campus Aurora, CO United States; 4 Division of Cardiology University of Colorado Anschutz Medical Campus Aurora, CO United States; 5 Adult and Child Consortium for Outcomes Research and Delivery Science University of Colorado Anschutz Medical Campus Aurora, CO United States; 6 Department of Biostatistics and Informatics Colorado School of Public Health Aurora, CO United States

**Keywords:** dementia, cognitive impairment, firearm, decision aid, caregivers, safety, feasibility, pilot trial, Alzheimer disease, caregiver support

## Abstract

**Background:**

Firearms are common in the households of persons with Alzheimer disease and related dementias (ADRD). Safety in Dementia (SiD) is a free web-based decision aid that was developed to support ADRD caregivers in addressing firearm access.

**Objective:**

We aimed to evaluate the feasibility and acceptability of SiD among a web-based sample of ADRD caregivers.

**Methods:**

SiD was tested in 2 phases by using participants who were recruited from a web-based convenience sample (Amazon Mechanical Turk participants). In phase 1, caregivers were randomized to view either the intervention (SiD) or the control (Alzheimer’s Association materials), and the blinding of participants to the study arms was conducted. In phase 2, caregivers of individuals with ADRD and firearm access were recruited; all of these participants viewed the firearm section of SiD. In both phases, participants viewed SiD independently for as long as they wanted. Measures for evaluating decision-making and SiD acceptability were used, and these were assessed via a self-administered web-based questionnaire.

**Results:**

Participants were recruited for phases 1 (n=203) and 2 (n=54). Although it was feasible to collect the study outcome data in a web-based format, in phase 1, there were no significant differences between SiD and the control in terms of decision-making and self-efficacy. The majority (137/203, 67.5%) of phase 1 participants spent between 5 and 10 minutes reviewing the resources. In phase 2, 61% (33/54) of participants spent 5 to 10 minutes viewing the firearm section, and 31% (17/54) spent 10 to 20 minutes viewing this section. Usability and acceptability were high across the phases.

**Conclusions:**

SiD represents a new resource for promoting safety among people with dementia, and high acceptability was achieved in a pilot trial. In this sample, SiD performed similarly to Alzheimer’s Association materials in supporting decision-making and self-efficacy.

## Introduction

Most firearm deaths among older adults are the result of suicide, but memory and behavior changes resulting from Alzheimer disease and related dementias (ADRD) have raised safety concerns among care partners and others. Dementia safety guidelines [1,2] recommend limiting access to firearms or other potentially dangerous items, but existing resources [3,4] have not adequately addressed logistics such as legal considerations. A recent large survey found that many ADRD caregivers were open to counseling and resources, but only 5% reported ever having a health care provider address firearm safety [5].

We previously created the web-based Safety in Dementia (SiD) decision aid [6,7] to support care partners. SiD guides users through questions, such as those about preferences for in-home storage versus out-of-home storage or how a person with ADRD may react to no longer having access to firearms. SiD’s sections were designed to help users find options that best matched their preferences, values, and situations. In other complex scenarios, decision aids have increased knowledge and decreased feelings of conflict, passivity, and apprehension [8].

Herein, we describe a pilot study for assessing the feasibility and acceptability of SiD among a web-based sample of caregivers. We sought to examine the feasibility of collecting outcome data and the acceptability of the tool in preparation for a future full-scale randomized trial. Although SiD situates firearm access within the context of other safety considerations (eg, driving and household safety) [6], we focused this evaluation on the firearm component of SiD.

## Methods

### Study Design and Population

We evaluated SiD in a 2-phase study by using samples from the Amazon Mechanical Turk (MTurk) platform [9]. MTurk is a web-based crowdsourcing platform where individuals complete tasks in exchange for digital currency. Eligible participants were English-speaking, US-based, adult users of MTurk (aged ≥18 years) who self-identified as informal caregivers of someone with ADRD who was not living in a nursing home or another facility that provided 24-7 care and supervision. Potentially eligible participants had to choose the correct definition of dementia as a check of their caregiver identity and attention (ie, to determine whether they were paying attention) [10]. Each participant completed 2 additional attention checks while taking the survey. Participants viewed study information for informed consent and were compensated with US $4.00. This amount was in line with the compensation amounts for comparable MTurk tasks. MTurk participants’ identities were not known to the study team.

In phase 1, participants were randomized (1:1) to view either the intervention (SiD) or control (static, web-based Alzheimer’s Association materials [3]). Participants were blinded to the study arms and could navigate through the study websites for as long as they wanted and in whatever way they wanted. They were asked to choose 1 section (the firearm, driving, or home safety sections) that was the “most meaningful in [their lives] right now” as caregivers and answer related questions.

After exceeding the target recruitment size for the pilot randomized trial (phase 1), we adjusted the eligibility criteria to specify that the person with ADRD must have access to at least 1 firearm (phase 2). This change was made to allow for the collection of additional focused feedback on the firearm section, and all caregiver participants in phase 2 were directed to view the firearm section of the SiD website. The SiD website content was frozen during this study, and no changes were made until after this study was completed. This study was approved by the Colorado Multiple Institutional Review Board, which waived the need for written informed consent.

### Measures

Web-based, self-administered questionnaires in Qualtrics (Qualtrics International Inc) were used to assess the characteristics of participants and the people with dementia for whom they provided care.

We assessed the feasibility of collecting data on key efficacy outcome measures from the Ottawa Decision Support Framework [11], which addresses decisional needs (eg, knowledge, conflicts, and personal values) that affect decisional quality (ie, the degree to which decisions align with values). The 10-item Preparation for Decision-Making Scale [12] uses Likert response options, and higher scores represent greater preparedness; we excluded the item on preparation for follow-up with a physician [12]. The Decision Self-Efficacy Scale [13] measures an individual’s self-confidence in their decision-making ability. The Stage of Decision-Making Scale uses a 6-point Likert scale and includes responses that range from “haven’t begun to think about the choices” to “have already made a decision and am unlikely to change my mind” [14]. Efficacy measures were administered after participants viewed SiD or the control; the Stage of Decision-Making Scale was also administered before participants viewed the study materials.

To analyze tool acceptability, we used the Ottawa Acceptability Scale [15] to assess the study materials’ balance in tone, the clarity of information, helpfulness, and the likelihood of participants recommending the study materials to others. Additional questions were used to assess tool usability and allowed for free-text feedback.

### Analysis

Quantitative survey data were analyzed by using descriptive statistics. Continuous variables were summarized with means and SDs (or with medians and quartiles when a group had a sample size of <10). Categorical variables were summarized with frequencies and percentages. Differences in measures between the control and SiD arms in phase 1 and between the phase 1 and phase 2 cohorts were tested with 2-sample two-tailed *t* tests for continuous variables and Fisher exact tests for categorical variables due to the small sample sizes in some groups. All phase 1 comparisons were conducted based on the intention-to-treat assignment to each study arm.

## Results

Between March and August 2020, 257 MTurk individuals participated in this study; we excluded 6 individuals who did not complete the questionnaires. In phase 1, caregivers were randomized to view either the SiD (n=98) or the control (n=105); there were no significant differences in the characteristics of participants or people with dementia (Table 1). The median age was 35 years (IQR: 15 years). Most participants were female (132/203, 65%) and White (157/203, 77.3%), and 11.8% (24/203) of participants were Hispanic. Of the 203 participants, 61 (30%) reported owning ≥1 firearm. Most participants (137/203, 67.5%) lived with the person with dementia for whom they provided care. Further, one-fifth (45/203, 22.2%) of participants reported that the person with dementia lived in a home with a firearm, and nearly 10% (18/203, 8.9%) reported that the person with dementia owned ≥1 firearm. In phase 1, participants (n=203) could choose which sections of SiD to review; of the 98 participants in the SiD group, 69 (70.4%) chose the “home safety” section, 14 (14.3%) chose the “firearms” section, and the remaining 15 (15.3%) chose the “driving” section.

In phase 2, 54 participants were enrolled. Compared to those in phase 1, phase 2 participants were more likely to be male, people of color, and Hispanic and care for individuals with less severe dementia (Table 1). In phase 2, 63% (34/54) of participants reported that the person with dementia lived in a home with ≥1 firearm, and nearly half (23/54, 43%) reported that the person with dementia owned ≥1 firearm.

**Figure 1:**
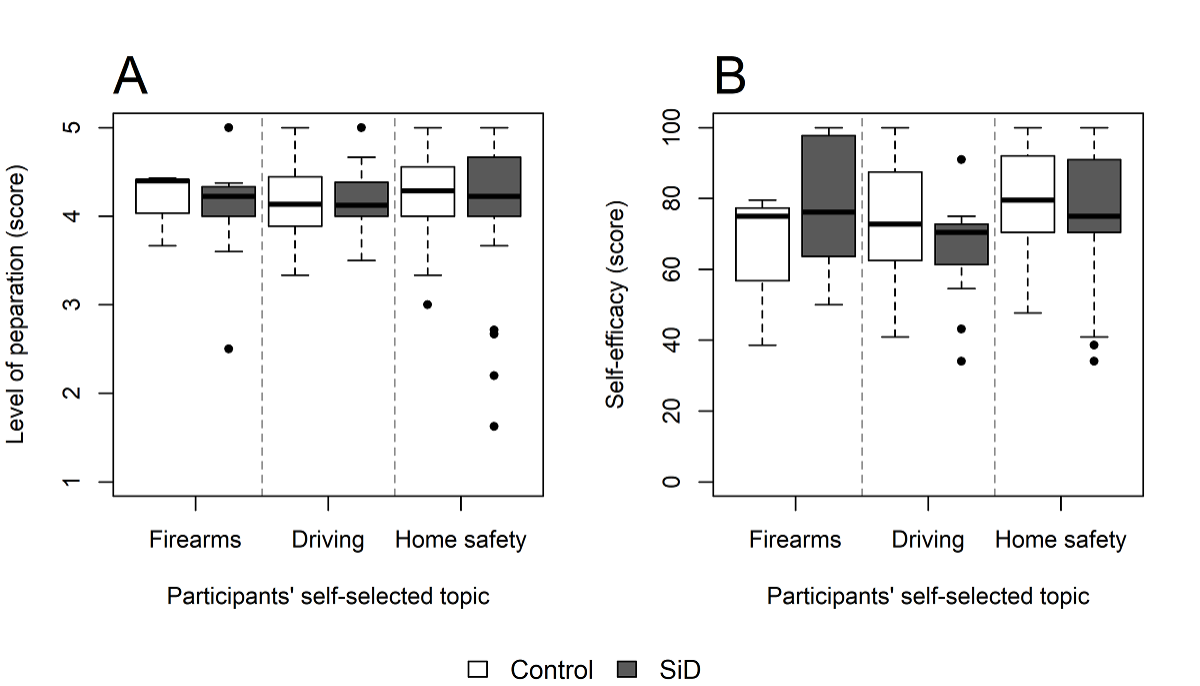
Plots showing the distribution of scores for participants' (A) preparation for decision-making and (B) decision self-efficacy. The results for each randomized group are stratified by participants' self-selected topic (phase 1: n=203). Bars represent the 25th and 75th percentiles. In the Preparation for Decision Making Scale, higher scores represent greater preparedness. In the Decision Self-Efficacy Scale, transformed scores range from 0 (extremely low) to 100 (extremely high self-efficacy). SiD: Safety in Dementia.

Overall, in phase 1, participants’ reported preparedness for decision-making and decision self-efficacy were both high, with no significant differences between the SiD and control groups (Figure 1). The median preparedness score for decision-making was also high in phase 2 (median 4.0; IQR 3.9-4.3; scale: range 1-5), as was the decision self-efficacy score (median 68.2; IQR 57.4-79.5; scale: range 0-100). The Stage of Decision-Making Scale scores, which were measured before and after viewing SiD or the control, did not significantly change in any group (Table 2).

**Table 1:** Participants’ characteristics stratified by study phase (N=257).^a^

Characteristics				Phase 1										Phase 2			
				Total (n=203)		Control group (n=105)		SiD^b^ group (n=98)		*P* value (control group vs SiD group)			Total (n=54)			*P* value (phase 2 total vs phase 1 total)	
Age (years), mean (SD)				36.6 (11.9)		36.0 (12.0)		37.3 (11.9)		.47			38.6 (13.7)			.34	
**Sex, n (%)**											.24						.03
	Male			71 (35)		41 (39)		30 (30.6)					28 (51.9)				
	Female			132 (65)		64 (61)		68 (69.4)					26 (48.1)				
**Race, n (%)**											.44						.07
	White			157 (77.3)		84 (80)		73 (74.5)					37 (68.5)				
	Black			16 (7.9)		6 (5.7)		10 (10.2)					6 (11.1)				
	Asian			15 (7.4)		9 (8.6)		6 (6.1)					4 (7.4)				
	American Indian or Alaska Native			4 (2)		2 (1.9)		2 (2)					6 (11.1)				
	Biracial			8 (3.9)		4 (3.8)		4 (4.1)					1 (1.9)				
Hispanic ethnicity, n (%)				24 (11.8)		14 (13.3)		10 (10.2)		.52			17 (31.5)			<.001	
**Highest level of education completed, n (%)**											.22						.02
	≤High school diploma			24 (11.8)		16 (15.2)		8 (8.2)					2 (3.7)				
	Some college			67 (33)		30 (28.6)		37 (37.8)					19 (35.2)				
	College diploma			84 (41.4)		42 (40)		42 (42.9)					17 (31.5)				
	≥Graduate training			28 (13.8)		17 (16.2)		11 (11.2)					16 (29.6)				
**Census region of residence, n (%)**											.63						.60
	Northeast			35 (17.2)		19 (18.1)		16 (16.3)					6 (11.1)				
	Midwest			41 (20.2)		18 (17.1)		23 (23.5)					13 (24.1)				
	South			88 (43.3)		49 (46.7)		39 (39.8)					22 (40.7)				
	West			39 (19.2)		19 (18.1)		20 (20.4)					13 (24.1)				
**Number of firearms personally owned, n (%)**											.24						<.001
	0			142 (70)		75 (71.4)		67 (68.4)					18 (33.3)				
	1			25 (12.3)		16 (15.2)		9 (9.2)					17 (31.5)				
	2-5			32 (15.8)		12 (11.4)		20 (20.4)					13 (24.1)				
	6 or more			4 (2)		2 (1.9)		2 (2)					6 (11.1)				
**Type of firearms owned (>1 response allowed), n (%)**																	
	Handgun, pistol, or revolver			53 (86.9)		26 (86.7)		27 (87.1)		>.99			26 (72.2)			.57	
	Rifle or long gun			26 (42.6)		10 (33.3)		16 (51.6)		.20			11 (30.6)			.65	
	Shotgun			25 (41)		12 (40)		13 (41.9)		>.99			14 (38.9)			>.99	
Zarit Caregiver Scale (6-question form)^c^ score, mean (SD)				9.8 (4.8)		10.0 (5.1)		9.7 (4.5)		.62			11.6 (4.6)			.02	
**Relationship with person with dementia, n (%)**											.44						.63
		Spouse or partner	13 (6.4)		7 (6.7)		6 (6.1)					6 (11.1)					
		Parent or stepparent	85 (41.9)		47 (44.8)		38 (38.8)					20 (37)					
		Other relative	88 (43.3)		46 (43.8)		42 (42.9)					22 (40.7)					
		Friend, neighbor, or coworker	10 (4.9)		3 (2.9)		7 (7.1)					3 (5.6)					
		Person cared for as part of work	7 (3.4)		2 (1.9)		5 (5.1)					3 (5.6)					
Lives with person with dementia, n (%)				137 (67.5)		68 (64.8)		69 (70.4)		.45			38 (70.4)			.75	
**Frequency of in-person contact (if participant does not live with person with dementia), n (%)**											.16						.93
		Daily	18 (27.3)		7 (18.9)		11 (37.9)					4 (25)					
		A few times per week	34 (51.5)		19 (51.4)		15 (51.7)					8 (50)					
		A few times per month	10 (15.2)		7 (18.9)		3 (10.3)					2 (12.5)					
		Once per month or less	4 (6.1)		4 (10.8)		0 (0)					2 (12.5)					
**Dementia severity, n (%)**																	
		≥Moderate memory loss	144 (70.9)		73 (69.5)		71 (72.4)		.76			26 (48.1)			.002		
		≥Usually does not recognize close family members	87 (42.9)		42 (40)		45 (45.9)		.40			24 (44.4)			.88		
		≥Moderate difficulty making decisions	143 (70.4)		73 (69.5)		70 (71.4)		.88			30 (55.6)			.05		
**Area where person with dementia lives, n (%)**											.82						.16
		Urban	63 (31)		32 (30.5)		31 (31.6)					24 (44.4)					
		Suburban	103 (50.7)		52 (49.5)		51 (52)					24 (44.4)					
		Rural	37 (18.2)		21 (20)		16 (16.3)					6 (11.1)					
**Activities of person with dementia, n (%)**																	
		Lives in home with firearm	45 (22.2)		27 (25.7)		18 (18.4)		.24			34 (63)			<.001		
		Drives a car	32 (15.8)		20 (19)		12 (12.2)		.25			17 (31.5)			.02		
		Spends time alone at home	104 (51.2)		54 (51.4)		50 (51)		>.99			34 (63)			.17		
**Has ever had concerns that the person with dementia might not be safe when performing the following (response: yes), n (%)**																	
		Having firearm access	49 (24.1)		24 (22.9)		25 (25.5)		.74			26 (48.1)			.001		
		Driving	98 (48.3)		52 (49.5)		46 (46.9)		.78			33 (61.1)			.13		
		Having unsupervised access to items or areas at home	165 (81.3)		88 (83.8)		77 (78.6)		.37			30 (55.6)			<.001		
**Number of firearms owned by person with dementia, n (%)**											.93						<.001
		0	185 (91.1)		95 (90.5)		90 (91.8)					30 (55.6)					
		1	6 (3)		3 (2.9)		3 (3.1)					15 (27.8)					
		2-5	12 (5.9)		7 (6.7)		5 (5.1)					5 (9.3)					
		6 or more	0 (0)		0 (0)		0 (0)					3 (5.6)					
		Not sure/missing	0 (0)		0 (0)		0 (0)					1 (1.9)					
**Type of firearms owned by person with dementia (>1 response allowed)**																	
		Handgun, pistol, or revolver	13 (72.2)		6 (60)		7 (87.5)		.31			16 (66.7)			.70		
		Rifle or long gun	7 (38.9)		6 (60)		1 (12.5)		.07			7 (29.2)			.06		
		Shotgun	3 (16.7)		1 (10)		2 (25)		.56			10 (41.7)			.13		

^a^Counts may not add up to the totals due to missing data (ie, results for items with <5% of the data are not shown).

^b^SiD: Safety in Dementia.

^c^The 6-item short version of the Zarit Caregiver Scale has Likert response options that range from 0 (never) to 4 (nearly always); higher cumulative scores represent greater burden [16].

**Table 2:** Changes in stages of decision-making stratified by study phase (N=257).^a^

Stage of decision-making	Phase 1		Phase 2
	Control group (n=105), n (median score; quartile, 3rd quartile)	SiD^b^ group (n=98), n (median score; quartile, 3rd quartile)	SiD group (n=54), n (median score; quartile, 3rd quartile)
Firearms (preintervention)	2 (2.5; 1.8, 3.2)	8 (3.0; 3.0, 6.0)	42 (4.0; 3.0, 5.0)
Firearms (postintervention)	2 (4.5; 4.2, 4.8)	12 (3.0; 2.8, 3.2)	48 (3.0; 2.0, 4.2)
Driving (preintervention)	19 (3.0; 2.5, 6.0)	15 (4.0; 3.0, 5.0)	—^c^
Driving (postintervention)	22 (3.0; 3.0, 5.8)	15 (3.0; 3.0, 5.0)	—
Home safety (preintervention)	68 (4.0; 3.0, 5.2)	56 (5.0; 3.0, 6.0)	—
Home safety (postintervention)	71 (4.0; 3.0, 6.0)	61 (5.0; 3.0, 5.0)	—

^a^Excludes missing data and those who answered “not an issue.”

^b^SiD: Safety in Dementia.

^c^Not available.

Usability and acceptability were high across groups, including both the SiD and control groups. The majority (137/203, 67.5%) of participants spent between 5 and 10 minutes reviewing the resources. Among those in phase 2, 61% (33/54) spent 5 to 10 minutes viewing the firearm section, and 31% (17/54) spent 10 to 20 minutes viewing the firearm section. A participant wrote:

I think that the firearm material was very informative and thorough. It gave good examples of real-life situations and how to handle decisions base[d] upon many different perspectives (ex who owns gun) within the household. I felt like it was a very good resource to be able to rely on.

With regard to the firearm section, 51% (36/71) of those who viewed it reported that it had the right amount of information, 83% (59/71) reported that most or all things were clear, 73% (52/71) reported that it was somewhat or very helpful, and 82% (58/71) reported that they would probably or definitely recommend it to others facing similar decisions or questions.

## Discussion

### Principal Results

SiD represents the first publicly available decision aid that addresses firearm access among people with dementia [6]. This trial demonstrated the feasibility of recruiting caregivers through MTurk and collecting efficacy outcome data. In the randomized phase, the interactive aid—SiD—performed similarly to the static Alzheimer’s Association materials in terms of its effects on decision-making and decision self-efficacy. Users of both resources may be more knowledgeable in and supportive of decision-making than people who are not directed to a resource, and this could be tested in future work. The phase 2 results indicated that ADRD care partners were willing to engage with the decision aid, found it useful for making decisions, and would recommend the resource to others.

The Veterans Health Administration has created guidance memoranda for clinicians on when and how to counsel veterans with dementia (and their caregivers) about safe firearm practices [4]. Some ADRD organizations have coordinated with firearm retailers to provide temporary storage options for ADRD caregivers who may need assistance in moving firearms from their homes [17]. Although these organizations have provided general guidance, SiD represents a practical tool for supporting decision-making. It can be used as a stand-alone resource for care providers, although it might also be integrated into counseling provided by care providers in health care or aging service organizations [18].

Quantitative and qualitative feedback resulted in the refinement of SiD. To make resources more accessible, we added a downloadable summary in each section. We revised the language to normalize the idea that solutions can take time and effort to enact. Further improvements to website navigation and flow (ie, restructuring the website to clarify where certain content was located) were made via consultations with a web developer.

### Limitations

The limitations of this pilot study include the fact that participants were predominantly non-Hispanic, White individuals. Existing data indicate that firearm ownership and suicide are more common among White individuals than among other racial and ethnic groups [19], but more diverse samples could reveal differences among populations. SiD has now been translated into Spanish to allow for future testing and use among broader populations. Further, MTurk participants may be a more technologically savvy population, and this may have inflated our results on the acceptability of a web-based tool. Larger-scale studies that examine effective dissemination strategies for reaching diverse populations as well as the effect that SiD has on key outcomes, such as injuries and caregiver well-being, are current research foci.

### Conclusions

Our pilot trial results suggest that SiD represents a practical, interactive tool that is usable and acceptable among ADRD caregivers. SiD seeks to frame critical decision points and present information in clear and digestible segments to make decisions more manageable and, consequently, more likely to be enacted [20]. Additional testing is needed to evaluate its effects on behavior changes and outcomes among both caregivers and people with dementia and to identify the best methods for disseminating SiD to diverse populations affected by ADRD.

## Appendix

Multimedia Appendix 1 CONSORT-eHEALTH checklist (V 1.6.1).

## Abbreviations

ADRD: Alzheimer disease and related dementias
MTurk: Amazon Mechanical Turk
SiD: Safety in Dementia
